# Improved Mobility and Quality of Life Through Physical Activity Management in a Stroke Patient With Peripheral Arterial Disease: A Case Report

**DOI:** 10.7759/cureus.84149

**Published:** 2025-05-15

**Authors:** Takashi Maeno, Izumi Matsumoto, Kazuhiro Harada

**Affiliations:** 1 Department of Rehabilitation, Yuseikai Midorigaoka Hospital, Takatsuki, JPN; 2 Graduate School of Health Science Studies, Kibi International University, Takahashi, JPN

**Keywords:** convalescent rehabilitation wards, health-related quality of life, multimorbidity, peripheral arterial disease, physical activity, stroke

## Abstract

Stroke and peripheral arterial disease (PAD) are common causes of impaired walking and reduced physical activity levels, both of which contribute to a decline in quality of life (QOL). Effective rehabilitation strategies that integrate functional training and physical activity management are essential for improving walking ability and QOL in patients with such multimorbidity. This case report describes a 70-year-old woman with left hemiparesis due to branch atheromatous disease and PAD (Fontaine stage IIb). She participated in a comprehensive rehabilitation program that combined functional training and physical activity management using a triaxial accelerometer. The intervention included strength training, treadmill walking, and balance exercises conducted seven days per week for 60-120 minutes per day, targeting a Borg scale rating of 13-15 for lower limb fatigue. Physical activity management focused on reducing sedentary behavior (SB) and increasing light-intensity physical activity (LPA) and daily step counts through goal setting and real-time feedback from the accelerometer. After 39 days, her six-minute walk distance increased to 190 meters, walking speed improved to 1.11 m/s, and initial claudication distance extended to 150 meters. Her daily step count rose to 3,204, LPA increased to 381 minutes per day, and SB decreased to 479 minutes per day. Health-related QOL, assessed using the Walking Impairment Questionnaire (WIQ) and the Vascular Quality of Life Questionnaire (VascuQOL), also showed marked improvement. This case suggests that intensive functional training combined with high-frequency physical activity management may be effective for improving mobility and QOL in patients with multimorbidity in convalescent rehabilitation wards.

## Introduction

In older adults with chronic diseases and disabilities, physical activity positively influences a range of health-related outcomes. To obtain these benefits, current guidelines recommend engaging in at least 150-300 minutes of moderate-intensity aerobic activity or 75-150 minutes of vigorous-intensity aerobic activity per week. Additionally, reducing prolonged sedentary time and replacing it with light physical activity can further enhance health outcomes [1].

Among stroke survivors, physical activity levels are associated with walking independence [2]. However, many patients are unable to meet recommended activity levels due to physical impairments, such as motor paralysis and balance dysfunction, following a stroke [3]. Therefore, both physical function and activity levels must be improved in parallel during convalescent rehabilitation.

Peripheral arterial disease (PAD), typically caused by atherosclerosis, results in reduced blood flow due to arterial narrowing or occlusion in the lower limbs. This condition often leads to intermittent claudication, characterized by muscle weakness and walking-induced pain. Intermittent claudication significantly impairs mobility, thereby limiting physical activity [4]. Furthermore, individuals with PAD frequently exhibit sarcopenia, defined as reduced skeletal muscle mass, strength, and physical function, and are consequently less active [5]. Notably, patients with PAD are known to exhibit prolonged sedentary behavior (SB), which is associated with lower survival rates, as reported in a previous study [6]. Despite the importance of physical activity in preventing cerebrovascular and cardiovascular complications, effective intervention strategies to increase activity in patients with both PAD and stroke remain unclear.

In patients with multiple chronic conditions, physical activity has been linked to improved prognosis and extended life expectancy [7]. In this study, the term "multimorbidity" specifically refers to the combination of stroke, PAD, hypertension, and dyslipidemia. Enhancing activity levels may slow disease progression and improve both quality of life (QOL) and clinical outcomes [8]. While previous studies have addressed the impact of multimorbidity on QOL and physical activity, there are few interventional reports on activity monitoring or management in convalescent rehabilitation wards for patients with complex multimorbidity, including stroke and PAD. This report presents a case of a stroke patient with PAD who underwent convalescent rehabilitation, showing improvements in physical function, activity levels, and QOL through the integration of physical activity management using an activity monitor in conjunction with functional training.

## Case presentation

The patient was a woman in her 70s (height: 152 cm; BMI: 18.6 kg/m²) who presented with left hemiplegia due to a branch atheromatous disease (BAD). The Fugl-Meyer Assessment for the lower extremity (FMA-LE) score was 30 out of 34, indicating mild motor paresis and the presence of ataxic symptoms. There was no significant motor impairment in the left upper extremity. She was admitted to convalescent rehabilitation wards for the purpose of returning home (day 0). Her comorbidities included PAD, dyslipidemia, and hypertension. Prior to admission, she lived independently with her husband and ambulated without assistance. However, she experienced intermittent claudication with heaviness in both lower limbs, particularly the right, which occurred after walking approximately 100 meters. Her primary complaint was as follows: "My right leg feels heavy and I can't walk for long. I also feel insecure about my left leg. I don't want to walk."

**Figure 1:**
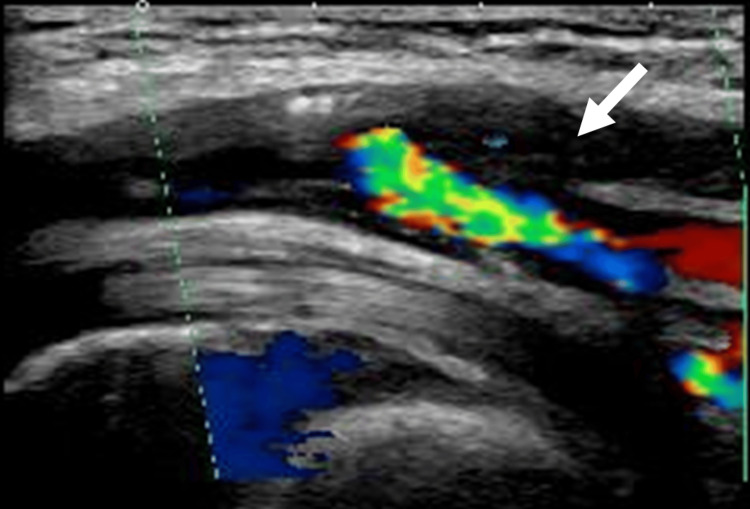
Color Doppler ultrasound image Color Doppler ultrasound image showing the right SFA, with the upper bifurcated vessel displaying no detectable color flow signal, indicating complete occlusion due to calcification (white arrows). SFA: superficial femoral artery

Her medications included aspirin, prasugrel hydrochloride, amlodipine besilate, and rosuvastatin calcium. On day 12 post-admission, an ankle-brachial index (ABI) test revealed abnormally low values of 0.41 in the right lower limb and 0.52 in the left. Lower limb ultrasound demonstrated calcification and arterial occlusion, particularly in the right superficial femoral artery (Figure 1). PAD was diagnosed based on clinical symptoms, ABI findings, and lower limb ultrasound findings, corresponding to Fontaine stage IIb, which is characterized by intermittent claudication after walking a short distance.

The initial physical function assessment on day 0 included FMA-LE, handgrip strength (HGS), Short Physical Performance Battery (SPPB), walking speed, six-minute walk distance (6MWD), initial claudication distance (ICD), pain recovery time (RT), and gait pattern analysis. Body composition was assessed using bioelectrical impedance analysis (InBody S10, InBody Co., Ltd., South Korea) to determine appendicular skeletal muscle mass index (ASMI) and extracellular water-to-total body water ratio (ECW/TBW). The patient demonstrated reduced function, with HGS at 17 kg, gait speed of 0.59 m/s, SPPB score of 7, and ASMI of 4.94 kg/m². According to the Asian Working Group for Sarcopenia criteria, she was diagnosed with severe sarcopenia [9] (Table 1).

**Table 1:** Changes in measurement variables FMA-LE: Fugl-Meyer Assessment for the lower extremity; HGS: handgrip strength; SPPB: Short Physical Performance Battery; 6MWD: six-minute walk distance; ICD: initial claudication distance; RT: recovery time; ASMI: appendicular skeletal muscle mass index; ECW/TBW: extracellular water/total body water ratio; SB: sedentary behavior; LPA: light-intensity physical activity; MVPA: moderate to vigorous physical activity

Measurement variables/hospital days	Initial evaluation (day 0)	Progress I (days 13-15)	Interim evaluation 1 (day 16)	Progress II (days 16-21)	Interim evaluation 2 (day 22)	Progress III (days 22-27)	Progress IV (days 28-34)	Final evaluation (day 39)
FMA-LE (points)	30	-	32	-	33	-	-	33
HGS (kgf)	17	-	17.5	-	17.5	-	-	18
SPPB (points)	7 (3-2-2)	-	9 (4-3-2)	-	10 (4-4-2)	-	-	12 (4-4-4)
Walking speed (m/s)	0.59	-	0.83	-	1.00	-	-	1.11
6MWD (m)	150	-	160	-	180	-	-	190
ICD (m)	70	-	90	-	130	-	-	150
RT (min)	5.5	-	5	-	4	-	-	4
Gait pattern	Wheelchair	Walker	Walker	Walker	Walker/stick	Stick	Stick	No walking aids
ASMI (kg/m^2^)	4.94	-	-	-	5.11	-	-	5.01
ECW/TBW	0.399	-	-	-	0.398	-	-	0.399
Wearing time (min)	-	854	-	863	-	865	871	-
Time in SB (min)	-	493	-	488	-	457	479	-
Time in SB (%)	-	57.7	-	56.5	-	52.8	55	-
≥30 min sedentary bout duration (min)	-	172	-	166	-	123	105	-
Percentage SB time in ≥30 min SB bouts (%)	-	34.9	-	34	-	26.9	21.9	-
≥60 min sedentary bout duration (min)	-	41	-	0	-	21	9	-
Percentage SB time in ≥60 min SB bouts (%)	-	8.3	-	0	-	4.6	1.9	-
Time in LPA (min)	-	353	-	361	-	398	381	-
Time in LPA (%)	-	41.3	-	41.8	-	46	43.7	-
Time in MVPA (min)	-	8	-	12	-	9	11	-
Time in MVPA (%)	-	0.9	-	1.4	-	1	1.3	-
Step counts (steps/day)	-	1983	-	1715	-	2305	3204	-

On day 12, a triaxial accelerometer (HJA-750C Active Style Pro, Omron Healthcare Co., Ltd., Muko, Kyoto, Japan) was introduced to monitor physical activity as the patient had achieved sufficient motor recovery and clinical stability to walk independently with a walker.

The primary evaluation parameters included the following: daily step count: total number of steps per day, automatically recorded by the accelerometer and averaged over valid wear days; SB: ≤1.5 METs; light-intensity physical activity (LPA): 1.6-2.9 METs; and moderate to vigorous physical activity (MVPA): ≥3.0 METs. SB time, LPA time, and MVPA time were defined as the total daily duration spent in each intensity category (in minutes/day). The following metrics were calculated to assess physical activity patterns: time-based averages (min/day) for SB, LPA, and MVPA, percent of wear time (%) for each category, and sedentary bouts, defined as continuous SB ≥30 minutes, with proportion of bout durations (≥30 or ≥60 min) calculated relative to SB time and total wear time.

The rehabilitation program consisted of daily functional training, resistance exercises, standing and walking practice, treadmill walking, and activities of daily living (ADL) training, performed seven days per week for 60-120 minutes per day. While specific daily session breakdowns were not recorded, the program was structured progressively to increase task difficulty over time. Exercise intensity was prescribed at a Borg rating of 13-15 (lower limb fatigue). Physical activity management involved goal-setting, feedback via the accelerometer, use of a daily step diary, self-exercise guidance, and scheduling adjustments based on daily activity fluctuations.

During progress I (days 13-15), prolonged SB time and SB bout duration were observed. To prevent stroke recurrence and PAD progression, we emphasized reducing sedentary time and replacing it with LPA. The patient was encouraged to use a pedometer and increase daily step count.

**Figure 2:**
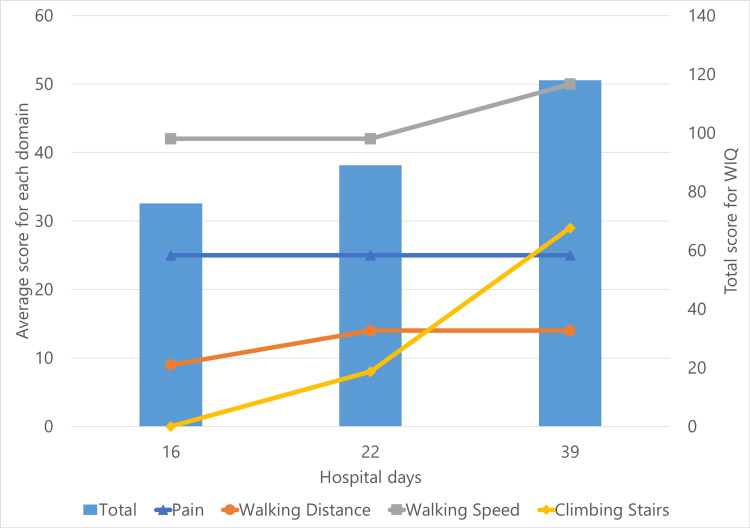
Changes in the WIQ score over time The left axis indicates the score for each domain and the right axis shows the total score. This figure illustrates the changes over time in the WIQ score, a functional quality of life assessment for patients with PAD and intermittent claudication. The score was measured at three time points: interim evaluation 1 (day 16), interim evaluation 2 (day 22), and final evaluation (day 39). Overall, the WIQ score showed a gradual improvement. Each domain, namely, walking distance, walking speed, and stair climbing, also demonstrated progressive gains. WIQ: Walking Impairment Questionnaire; PAD: peripheral arterial disease

**Figure 3:**
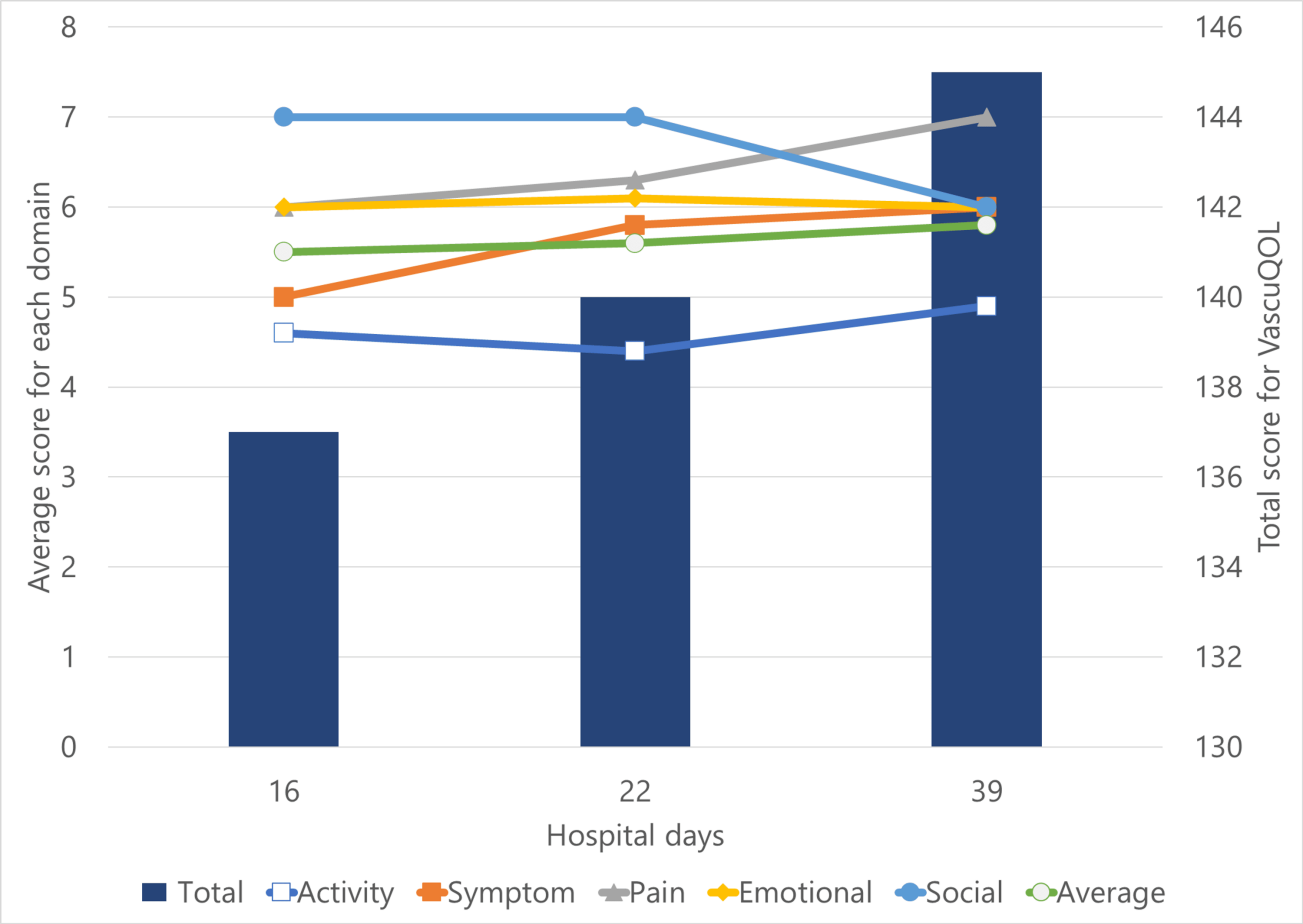
Changes in the VascuQOL score over time The left axis indicates the score for each domain and the right axis shows the total score. This figure illustrates the changes over time in the VascuQOL score, a disease-specific measure of quality of life in patients with PAD. The score was assessed at three time points: interim evaluation 1 (day 16), interim evaluation 2 (day 22), and final evaluation (day 39). Although some fluctuations were observed across individual domains, the total score demonstrated a gradual overall improvement. VascuQOL: Vascular Quality of Life Questionnaire; PAD: peripheral arterial disease

At interim evaluation 1 (day 16), the Walking Impairment Questionnaire (WIQ) and the Vascular Quality of Life Questionnaire (VascuQOL) were administered to assess functional and vascular QOL (Figure 2 and Figure 3). The WIQ score (total) was 76 points, and the VascuQOL score (total) was 137 points.

During progress II (days 16-21), although physical function improved, daily step count decreased while LPA time slightly increased and SB time decreased. Feedback was provided based on pedometer readings, and self-training in a standing position was recommended to improve activity levels. Therapy sessions were rescheduled to target periods of reduced activity.

At interim evaluation 2 (day 22), improvements were noted in gait speed and SPPB score (from 7 to 10). ASMI improved to 5.11 kg/m², and sarcopenia status improved from "severe" to "mild". WIQ and VascuQOL scores also showed upward trends.

In progress III (days 22-27), step counts further increased, SB time and its percentage decreased, and both LPA time and its percentage continued to rise. MVPA time remained unchanged. To further enhance activity intensity, we introduced walking drills and self-exercise targeting gait speed and cadence within both the physical therapy room and the ward. Stair climbing was also incorporated in preparation for discharge.

During progress IV (days 28-34), relative to progress I, SB time, SB bout time, and SB percentage decreased, while step count, LPA, MVPA, and their respective percentages increased.

At the final evaluation (day 39), FMA-LE score improved to 33, HGS to 18 kg, SPPB to 12, and walking speed to 1.11 m/s. The patient no longer met the criteria for sarcopenia. Improvements were also seen in 6MWD (190 m), ICD (150 m), and RT (four minutes). WIQ score (total) was 118 points, showing improvement in each domain. VascuQOL score (total) improved to 145 points, but the degree of improvement varied across each domain. The patient was discharged home on day 45.

## Discussion

Functional training combined with physical activity management using a triaxial accelerometer in convalescent rehabilitation wards led to improvements in physical activity levels, functional capacity, and QOL in a stroke patient with comorbid PAD, hypertension, and dyslipidemia.

Physical activity levels in stroke patients are significantly lower than those of healthy individuals [10]. One study reported that patients in the recovery phase of stroke average 5,535 steps per day [10]. Among patients with PAD and intermittent claudication, the average step count is approximately 3,586 steps per day, with SB lasting up to seven hours daily, substantially lower than in healthy individuals [11]. In the present case, baseline physical activity levels were also below those reported in previous studies.

Prior research has shown that in patients with chronic diseases and high SB time, physical activity can be effectively increased through strategies such as self-monitoring of step count, goal setting, step diaries, and individualized guidance [12]. In this case, similar approaches applied at high frequency within convalescent rehabilitation wards contributed to increased step count and reduced SB time.

In stroke survivors, improvements in basic mobility, such as sit-to-stand and stand-to-sit transitions, are associated with increases in LPA [13]. In patients with PAD and intermittent claudication, factors such as sarcopenia, muscle weakness, and reduced walking efficiency correlate with increased SB time and decreased LPA time [5,14,15]. The patient showed increased handgrip strength, improved gait speed and walk distance, higher SPPB scores, and improved ASMI, suggesting not only enhanced functional capacity but also a reduction in sarcopenia severity. In the current case, sarcopenia and exercise performance improved concurrently, suggesting that physical activity management helped mitigate functional decline caused by the disease. Given that individuals with PAD frequently exhibit sarcopenia and reduced physical activity [5], the observed improvement may reflect the positive impact of increased physical activity and rehabilitation on muscle function in this population.

Sex-specific differences have also been reported in PAD. Women tend to experience earlier onset of claudication, greater difficulty increasing exercise intensity, and lower WIQ scores compared to men [16]. The VascuQOL score has been shown to correlate strongly with the 6MWD [17]. Furthermore, individuals who accumulate more LPA tend to have better overall physical activity levels, functional capacity, and QOL than those with predominantly sedentary lifestyles [18]. Based on these findings, this patient likely had a predisposition toward declining physical function and activity levels. The WIQ score, a functional QOL assessment specific to intermittent claudication in PAD patients, showed improvement in all domains in parallel with exercise performance. However, the VascuQOL score, which includes not only physical function but also various other domains, showed varying degrees of improvement depending on the domain. This may be due to the coexistence of the condition felt during rehabilitation and the condition felt during daily life in the ward.

From a multimorbidity perspective, exercise-based rehabilitation has been shown to improve physical function, 6MWD, QOL, and cardiometabolic outcomes [19]. While greater numbers of comorbid conditions are associated with diminished QOL, increasing physical activity can help prevent disease progression and improve prognosis [8]. Although previous studies have described the negative impact of multimorbidity on physical activity and QOL, interventional approaches targeting this population remain limited. This case is novel in that it presents a structured, accelerometer-guided physical activity management approach within convalescent rehabilitation wards, addressing multiple coexisting conditions including stroke, PAD, sarcopenia, hypertension, and dyslipidemia.

As demonstrated in this case, reports focusing on the integration of physical activity management and functional training in patients with multimorbidity, such as stroke, PAD, dyslipidemia, and hypertension, are essential for informing rehabilitation strategies aimed at optimizing functional outcomes and preventing complications. Comprehensive patient assessment, health education, and individualized management are crucial components of care for individuals with multiple chronic conditions [20]. Based on these insights, we believe that high-frequency physical activity management and personalized functional training in convalescent rehabilitation wards were effective in this case.

## Conclusions

For stroke patients with PAD, hypertension, and dyslipidemia, functional training in convalescent rehabilitation wards and physical activity management using a physical activity meter resulted in improvements in activity levels (step count, LPA, MVPA, and sedentary time), physical function, and QOL. It is important to advance physical activity management individually and gradually in line with improvements in physical function. For patients with multimorbidity, intensive functional training and high-frequency physical activity management in convalescent rehabilitation wards may be effective.
